# Radiomics and outcome prediction to antiangiogenic treatment in advanced gastroenteropancreatic neuroendocrine tumours: findings from the phase II TALENT trial

**DOI:** 10.1038/s44276-023-00010-0

**Published:** 2023-08-02

**Authors:** Marta Ligero, Jorge Hernando, Eric Delgado, Alonso Garcia-Ruiz, Xavier Merino-Casabiel, Toni Ibrahim, Nicola Fazio, Carlos Lopez, Alexandre Teulé, Juan W. Valle, Salvatore Tafuto, Ana Custodio, Nicholas Reed, Markus Raderer, Enrique Grande, Rocio Garcia-Carbonero, Paula Jimenez-Fonseca, Alejandro Garcia-Alvarez, Manuel Escobar, Oriol Casanovas, Jaume Capdevila, Raquel Perez-Lopez

**Affiliations:** 1 Radiomics Group, Vall d’Hebron Institute of Oncology (VHIO), Barcelona, Spain; 2 Oncology Department, Vall d’Hebron Institute of Oncology (VHIO), Barcelona, Spain; 3 Radiology Department, Vall d’Hebron University Hospital (VHUH), Barcelona, Spain; 4 Osteoncology and Rare Tumours Centre, Istituto Scientifico Romagnolo per lo Studio e la Cura dei Tumori (IRST), Meldola, Italy; 5 Units of Gastrointestinal and Neuroendocrine Tumours, European Institute of Oncology, Milan, Italy; 6 Oncology Department, Marques de Valdecilla University Hospital (IDIVAL), Santander, Spain; 7 Oncology Department, Catalan Institute of Oncology (ICO), L’Hospitalet de Llobregat (Barcelona), Spain; 8 University of Manchester and The Christie NHS Foundation Trust, Manchester, UK; 9 S.C. Sarcomi e Tumori Rari, Istituto Nazionale Tumori, IRCCS, Fondazione “G. Pascale”, Naples, Italy; 10 Oncology Department, La Paz University Hospital, Madrid, Spain; 11 Gartnavel Hospital, Beatson Oncology Centre, Glasgow, UK; 12 Department of Oncology and Internal Medicine, Medical University of Vienna, Vienna, Austria; 13 Oncology Department, MD Anderson Cancer Center, Madrid, Spain; 14 Oncology Department, Hospital Universitario 12 de Octubre, Imas12, UCM, Madrid, Spain; 15 Oncology Department, Central de Asturias University Hospital, Oviedo, Spain

## Abstract

**Background:**

More accurate predictive biomarkers in patients with gastroenteropancreatic neuroendocrine tumours (GEP-NETs) are needed. This study aims to investigate radiomics-based tumour phenotypes as a surrogate biomarker of the tumour vasculature and response prediction to antiangiogenic targeted agents in patients with GEP-NETs.

**Methods:**

In this retrospective study, a radiomics signature was developed in patients with GEP-NETs and liver metastases receiving lenvatinib. Patients were selected from the multicentre phase II TALENT trial (NCT02678780) (development cohort). Radiomics variables were extracted from liver metastases in the pre-treatment CT-scans and selected using LASSO regression and minimum redundancy maximum relevance (mRMR). Logistic regression and Cox proportional-hazards models for radiomics and combined radiomics with clinical data were explored. The performance of the models was tested in an external cohort of patients treated with sunitinib (test cohort). Associations between the radiomics score and vascularisation factors in plasma were studied using hierarchical clustering and Mann–Whitney *U* test.

**Results:**

A total of 89 patients were included in the study, 408 liver metastases were analysed. The CT-based radiomics signature was associated with clinical benefit in the development (training and validation sets) and test cohorts (AUC 0.75 [0.66–0.90], 0.67 [0.49–0.92] and 0.67 [0.43–0.91], respectively). The combined radiomics-clinical signature (including the radiomics score, Ki-67 index and primary tumour site) improved on radiomics-only signature performance (AUC 0.79 [95% CI 0.64–0.93]; *p* < 0.001).  A higher radiomics score indicated longer progression-free survival (hazard ration of 0.11 [0.03–0.45]; *p* = 0.002) and was associated with vascularisation factors (*p* = 0.01).

**Conclusions:**

Radiomics-based phenotypes can provide valuable information about tumour characteristics, including the vasculature, that are associated with response to antiangiogenics.

**Clinical trial registration:**

This is a study of the Lenvatinib Efficacy in Metastatic Neuroendocrine Tumours (TALENT) phase II clinical trial (NCT02678780).

## Background

Neuroendocrine tumours (NETs) are considered heterogeneous and complex to treat malignancies. Nevertheless, in the last two decades the treatment landscape for gastroenteropancreatic neuroendocrine tumours (GEP-NETs) has improved considerably, with an increase in available treatment strategies, making patient stratification and treatment selection more challenging. There is a wide range of effective therapies including somatostatin analogues, radiolabelled somatostatin analogues, molecular-targeted agents or chemotherapy [1–3]. Among them, antiangiogenic targeted therapies such as lenvatinib have demonstrated a high radiological response rate in these patients [4, 5]. Several clinical indicators have been considered as prognostic factors for NETs such as the Ki-67 proliferative index and disease staging [6]. However, there remains a clinical need to find predictive biomarkers of response to novel targeted therapies for achieving more precise patient selection [7].

Radiomics analysis allows for the extraction of quantitative data from routinely acquired medical images and correlation of imaging features with the underlying tumour characteristics including the tumour vasculature [8]. Radiomics opens a window of opportunity to develop new tools for improved prediction and response evaluation to novel treatment options. Current studies have been focused on applying radiomics for NET staging, grade characterisation and determination of alternative prognostic factors [9–19], but there is scarce data about radiomics to predict response to antiangiogenics in NETs [19]. Accounting for the emerging treatment strategies for NET patients, radiomics could also contribute in determining the best therapeutic approach for each patient.

The aim of this study was to develop and evaluate the performance of a computed tomography (CT)-based radiomics signature for tumour response prediction to antiangiogenic agents prior to treatment. Patients with advanced GEP-NETs enrolled in a multicentre phase II clinical trial conducted with the antiangiogenic targeted agent lenvatinib were included in the study [5]. To validate the signature, an external cohort of patients with pancreatic NET treated with another multikinase inhibitor with also proved antiangiogenic activity used in routine clinical practice, sunitinib, was studied. Secondarily, a multiphase model, that included information of both arterial and venous CT-image acquisitions, was explored to see whether this improved the performance for response prediction. Moreover, integration of the CT-based radiomic model and clinical data was also investigated in an attempt to improve the predictive value of this tool. We hypothesised that a CT-based radiomics phenotype can provide meaningful information about biology of NETs (including the tumour vasculature) and its susceptibility to respond to antiangiogenic treatment. The CT-based radiomics signature could be used to stratify patients by identifying those that are more likely to benefit from antiangiogenic targeted agents.

## Methods

The institutional review board approved this retrospective study. Informed consent for computational image analysis was waived.

All patients included in the clinical trial Lenvatinib Efficacy in Metastatic Neuroendocrine Tumours (TALENT) NCT02678780 [5] provided written informed consent.

### Study sample

The development cohort consisted of patients with GEP-NETs treated with the multikinase inhibitor lenvatinib in a multicentre, international, phase II clinical trial conducted from October 2015 to August 2020, identified as NCT02678780 (Supplementary Table 1 participant centres). The test cohort consisted of patients with pancreatic NETs treated with the multikinase inhibitor sunitinib as standard of care at the Vall d’Hebron Institute of Oncology (Barcelona, Spain) from October 2011 to September 2020.

Patients with GEP-NET liver metastasis and intravenous contrast-enhanced CT scans at baseline were included. Patients with artifacts at baseline CT scans and patients in which the clinical outcome could not be assessed due to toxicity or non-disease related death were excluded. A total of 408 liver metastases from 89 patients were included (46 men [52%] and 43 women [48%]), mean age 62 years (range 33–86).

### Image acquisition and radiomics analysis

All CT scans were acquired within 28-days before the treatment starting day. Contrast-enhanced images were obtained with 16- or 64-channel CT scanners (Siemens, Philips, GE Medical Systems, Toshiba, Agfa), 1–5 mm slice thickness and 100–120 kV of voltage (Supplementary Table 2). Up to six liver metastases per patient of at least 1 cm diameter were segmented using the semi-automatic segmentation tool of 3D Slicer (version 4.11.0; www.slicer.org; RRID:SCR_005619) [20] by a radiologist with more than 10 years of experience in oncological imaging (RPL). The tumours where segmented in the CT arterial or venous phase depending on the tumour contrast enhancement, selecting the phase in which tumours were better depicted (Supplementary Fig. 1). Combined models including information from both CT contrast-enhanced phases (arterial and venous) were also explored. Images were resampled to isotropic voxels of 1 × 1 × 1 mm^3^ by using spline interpolation. Hounsfield units were binarized to discrete values of 25 HU. CT-based radiomics features including first-order, shape and five gray-level texture matrices (Gray Level Co-occurrence Matrix [GLCM], Gray Level Dependence Matrix [GLDM)], Gray Level Run Length Matrix [GLRLM], Gray Level Size Zone Matrix [GLSZM], and Neighbouring Gray Tone Different Matrix [NGTDM]) were calculated in three dimensions using the Pyradiomics package (version 3.0.1) for Python (version 3.6.13 Python Software Foundation, Delaware, USA; RRID:SCR_008394), compliant with the Image Biomarker Standardisation Initiative guidelines [21]. To investigate the prediction power of a multiphase radiomics model, the arterial and venous CT-images were co-registered and the radiomics features from both acquisition phases were extracted (Supplementary Methods).

### Clinical data

Clinical data from the development cohort were collected from the TALENT clinical trial database where age, sex, primary tumour site, Ki-67 index, tumour burden, tumour grade and pre-treatment were registered. Vascular endothelial growth factor receptor 2 (VEGFR2) and angiopoietin 2 (ANG2) quantifications were obtained from patients with plasma samples. VEGFR2 and ANG2 plasma levels were determined by multiplex ELISA with a custom-made glass-slide sandwich Quantibody Array (RayBioTech, GA, USA). Regarding the test cohort, data were obtained from the electronic patient records. Clinical benefit was defined as achieving either a complete response (CR), partial response (PR) or stable disease (SD) by RECIST 1.1 for a duration exceeding the median progression-free survival (PFS) in the populations according to the phase II and III clinical trials [5, 22]. Therefore, patients treated with lenvatinib who progressed before 15.7 months and those treated with sunitinib who progressed before 11.4 months were considered as not achieving clinical benefit. Clinical data were used to develop a clinical only and a combined clinical-radiomics model.

### Modelling and statistical analysis

The development cohort was divided into training and validation sets (70–30%) balanced for outcome in both sets and then tested in an external cohort of patients with GEP-NETs (test cohort). As a standard method in machine learning modelling, the training set corresponded to the group of patients where the model was initially trained and the hyperparameters were fitted using cross-validation; in the validation set, we explored the model performance robustness; the final model generalisability was evaluated in the test cohort (i.e., the external validation cohort).

The median radiomics value of all the evaluated liver tumours per patient was implemented as feature aggregation method. We performed a two-step procedure for feature selection based on LASSO and minimum Redundancy Maximum Relevance (mRMR), these two have been shown to benchmark other feature selection methods [23]. Three-fold cross-validation was performed for LASSO hyperparameter tuning, choosing the model with one standard error from the best area under the curve (AUC). To avoid multicollinearity in the model features, mRMR was implemented to reduce the number of variables controlling for a variance inflation factor (VIF) < 2 and a Pearson square *R* < 0.5 [24]. Logistic regression model was performed including the selected features. The area under the curve (AUC) and 95% confidence interval (CI) (DeLong method) were computed from the receiver operating characteristic (ROC) curve and *p* values were assessed from Mann–Whitney *U* test. The decision threshold to compute sensitivity and specificity was defined by Youden’s index.

We explored the combination of the radiomics score with established prognostic factors in NETs, including Ki-67 index and primary tumour site. Since tumour grade is correlated with Ki-67 expression, it was not included as a separate factor in our analysis. Furthermore, the assessment of tumour burden, which can indicate different prognoses, was already incorporated into the radiomics model analysis. A logistic regression model including the radiomics score and non-correlated clinical variables (Ki-67 index, primary tumour site) was developed. Clinical data imputation was done using random forest. PFS associations with clinical variables and predictive radiomics scores were investigated using Cox Proportional-Hazard regression and Log-rank test. A multiphase model combining radiomics features from arterial and venous phases was also investigated (Supplementary Methods).

**Fig. 1:**
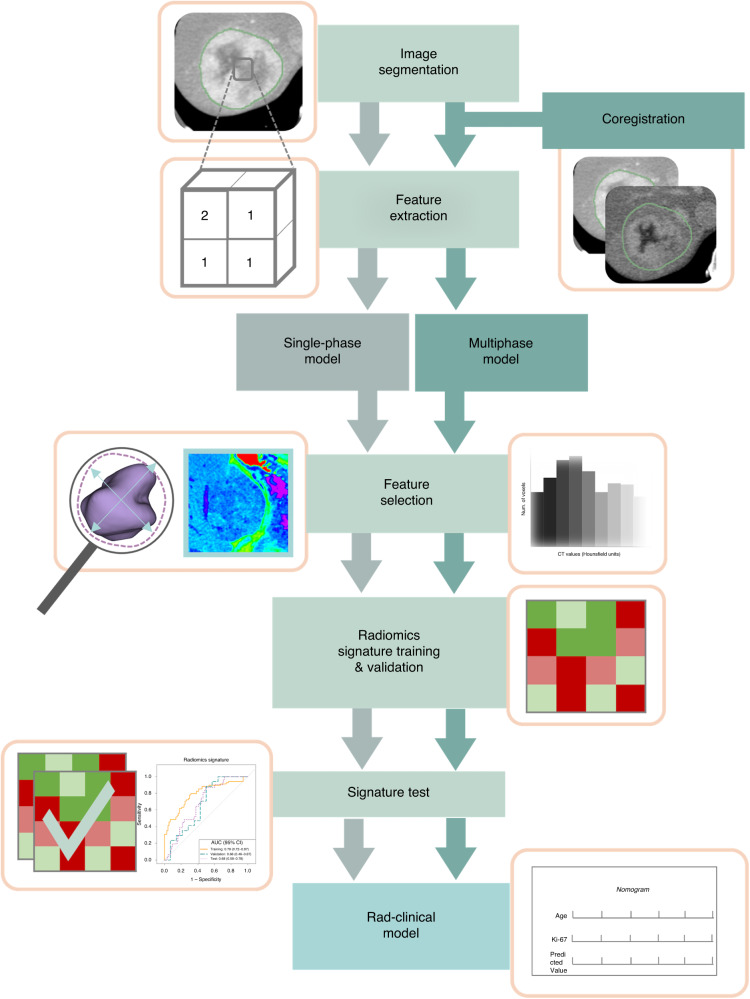
Radiomics analysis flowchart. The single-phase model is obtained from one of both acquisition CT phases (arterial or venous) of the development cohort, whereas the multiphase model included the co-registered images of the arterial and venous phases. Feature selection and model fitting was performed for both approaches. The model is validated in the test cohort and combined with clinical data to improve the predictive capacity.

Patients with both imaging and liquid biopsy were clustered based on vascularisation factors (including VEGFR2 and ANG2) using hierarchical clustering. Associations between radiomics score and vascularisation factors were studied using Mann–Whitney *U* test. The study workflow is summarised in Fig. 1.

## Results

### Study patient characteristics

From a total of 128 patients (88 from the development and 40 from the test cohorts), 15 patients without baseline contrast-enhanced CT scans or with artifacts in the area of interest were excluded, 19 without liver metastases or measurable lesions per RECIST 1.1 and 5 patients who presented toxicity before clinical outcome evaluation were also excluded. Eighty-nine patients were included in the radiomics analysis, 65 patients to build up the model (development cohort) and 24 for external validation (test cohort). Forty-four patients from the TALENT clinical trial had concomitant CT images and liquid biopsy samples.

For the exploratory analysis of combined information from both CT contrast-enhanced phases (arterial and venous), 65 patients (41 from the development and 24 from the test cohorts) had CT scans with both arterial and venous phases. A total of 282 tumours (5 [1–6] mean[range] lesion/patient) from the 65 patients of the development cohort; 34 women and 31 men were included in the final analysis. The median PFS was 14.9 [IQR 3.04–46.72] months; 46% (30/65) of patients presented clinical benefit (i.e., CR, PR or SD over the median PFS) and 54% (35/65) did not.

The model was validated in the test cohort including a total of 126 tumours from 24 patients (5 [1–6] mean[range] lesion/patient); 12 women and 12 men. The median PFS was 9.75 [IQR 2.90–29.47] months; 50% (12/24) of patients presented clinical benefit and 50% (12/24) did not.

In the development cohort, clinical benefit was defined as the combination of CR, PR or SD after 15.7 months, corresponding to the median PFS time in the TALENT clinical trial [5]. In the test cohort the cut-off for clinical benefit was defined at 11.4 months, corresponding to the median PFS time in the cohort [22].

Flow chart of the study population selection is shown in Supplementary Fig. 2. The population description is reported in Table 1.

**Table 1:** Population characteristics of the development and test cohorts.

Parameter	Development cohort (*n* = 65)	Test cohort (*n* = 24)
Mean age (years)^a^	60 ± 12(33–85)	63 ± 12 (38–86)
Sex		
Female	34 (52)	12 (50)
Male	31 (48)	12 (50)
Primary tumour site		
PanNET	24 (37)	23 (96)
GI-NET	33 (51)	1 (4)
Unknown	8 (12)	0 (0)
Pre-treatment		
Sunitinib	10 (15)	NA
Everolimus	19 (29)	NA
None	36 (55)	NA
Best response		
Partial response	24 (37)	6 (25)
Stable disease	40 (62)	15 (62)
Progressive disease	1 (1)	3 (13)
Mean Ki-67^a,b^	7.13 ± 5.87 (1.00–18.00)	21.96 ± 18.98 (1.30–55.10)
Grade^b^		
Grade 1	22 (34)	3 (12)
Grade 2	40 (61)	12 (50)
Grade 3	···	9 (38)
Unknown	3 (5)	
PFS (months)^c^	14.9 [3.04–46.72]	9.75 [2.90–29.47]
OS (months)^c^	33.7 [9.21–46.94]	12.1 [4.00–47.00]
Number of lesions	4 (1–6)	5 (1–6)
Mean hepatic tumour burden (dl)^a^	0.037 (0.001–2.209)	1.086 (0.007–1.054)
CT segmentation phase		
Arterial	23 (35)	7 (29)
Pancreatic NET	14 (21)	6 (25)
Gastrointestinal NET	9 (14)	1 (4)
Venous	42 (65)	17 (71)
Pancreatic NET	15 (23)	17 (71)
Gastrointestinal NET	27 (42)	···

Data in parentheses are percentages.

^a^Data are ± standard deviation and parentheses are range.

^b^Data presented has no information from three patients.

^c^Data are median [interquartile range].

### Predictive model development and testing

**Fig. 2:**
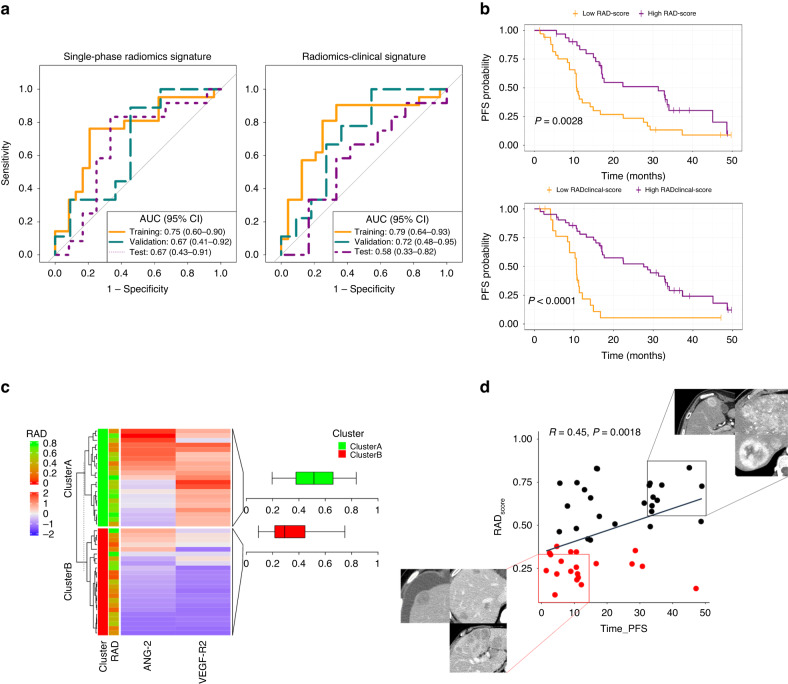
Response prediction performance and explainability. Performance of the radiomics and radiomics-clinical models to predict response to treatment using: **a** Receiver operating characteristic (ROC) curves for median progression-free survival (PFS) prediction and **b** Kaplan–Meier curves for PFS probability. Explainability of the radiomics signature: **c** Associations between vascular factor expression and radiomics score. The cluster A corresponds to highly vascularises tumours with high-radiomics score, while cluster B includes tumours with poor vascularisation and low-radiomics score. **d** Visualisation of tumours with high-radiomics score (i.e., more spherical, heterogenous and highly enhancing) and long PFS vs. low-radiomics score and short PFS. Spearman’s rank correlation analysis (*p* < 0.01).

LASSO regression lambda hyperparameter was set to 0.03 after cross-validation analysis with 13 radiomics features with non-zero coefficients. Six radiomics features were selected using mRMR method as the maximum number of variables to avoid multicollinearity in logistic regression. The final model included variables from first order, shape, and GLCM and GLRLM texture matrices (Table 2). The radiomics model combining the selected features predicted clinical benefit with an AUC of 0.75 [95% CI 0.60–0.90; *p* = 0.001] and 0.67 [95% CI 0.41–0.92; *p* = 0.115] in the training and validation sets, respectively. In the test cohort, the radiomics predicted response with an AUC of 0.67 [95% CI 0.43–0.91; *p* = 0.060] (Fig. 2a). Sensitivity and specificity for classifying patients with clinical benefit with an optimal Youden’s cut-off of 0.49 are described in Table 3. Internal cross-validation showed a mean AUC of 0.67 [0.50–0.87] (Supplementary Fig. 3). The radiomics model showed that tumour sphericity, heterogeneity assessed by GLCM Informational Measure of Correlation (imc), and enhancement were associated with clinical benefit (Fig. 2d). No significant differences in radiomics scores were found between patients previously treated with antiangiogenics and non-pre-treated patients (*p* = 0.33) (Supplementary Fig. 4). The radiomics score for predicting clinical benefit showed significant association with continuous PFS (HR 0.11[0.03–0.45]; *p* = 0.002) (Fig. 2b).

**Table 2:** Radiomics model coefficients.

Radiomics features	Coefficient [95% CI]
Shape flatness	−0.82 [−1.6–−0.05]
GLCM Imc1	0.31 [−0.47–1.09]
First order 90th percentile	0.37 [−0.29–1.04]
GLRLM Long Run Low Gray Level Emphasis	0.16 [−0.58–0.91]
First order skewness	0.34 [−0.35–1.03]
Shape sphericity	0.84 [0.05–1.63]

**Table 3:** Radiomics and radiomics-clinical model performance evaluation.

	AUC [95% CI]	*p* value	Accuracy (%)	Sensitivity (%)	Specificity (%)
Radiomics model					
Training	0.75 [0.60–0.90]	<0.001	78	76	79
Validation	0.67 [0.41–0.92]	0.114	55	55	56
Test	0.67 [0.43–0.91]	0.09	63	67	58
Radiomics clinical model					
Training	0.79 [0.64–0.93]	<0.001	78	90	67
Validation	0.72 [0.48–0.95]	0.12	70	45	100
Test	0.58 [0.33–0.82]	0.276	54	17	92

Table containing the area under the curve (AUC), accuracy, sensitivity and specificity for the Youden’s cut-off 0.49 for the radiomics-only model and 0.36 for the radiomics-clinical model.

The exploratory multiphase predictive model (combining information from the arterial and venous CT phases) did not improve the prediction capacity (AUC in test set 0.63 [0.39–0.87]) (Supplementary Fig. 5).

### Integrating radiomics and clinical data

The clinical model combining Ki-67 and primary tumour site showed poor performance in both the development (training 0.57 [0.40–0.74] and validation 0.56 [0.30–0.82]) and test sets (0.23 [0–0.46]). When combined with the radiomics score, the radiomics-clinical model predicted response with an AUC of 0.79 [0.64–0.93; *p* < 0.001] and 0.72 [0.48–0.95; *p* = 0.06] in the training and validation sets, respectively. In the test cohort, the combined radiomics-clinical score associated with response with an AUC of 0.58 [0.33–0.82; *p* = 0.27]. The sensitivity and specificity for classifying patients with clinical benefit with an optimal cut-off of 0.36 are described in Table 3. The ROC curves of the combined signature integrating radiomics-score and clinical variables were also computed (Fig. 2a). Tumour burden was not included in this integrative analysis because it was quantitatively included in the radiomics analysis. Tumour grade was also excluded due to correlation with Ki-67 index. The combined clinical-radiomics score for predicting clinical benefit showed significant association with continuous PFS (HR 0.12[0.04–0.43]; *p* = 0.001) (Fig. 2b). However, none of the clinical variables including Ki67, primary tumour site or tumour burden showed significant associations with PFS (*p* < 0.05) (Supplementary Fig. 6).

### Correlation of the CT-based radiomics score and tumour vasculature

The study population was clustered according to proangiogenic profiling factors, VEGFR2 and ANG2, by hierarchical clustering. Cluster A included 21 patients grouped as highly vascularised tumours showing higher values of VEGFR2 [median 18,784 (IQR 17,579–21,842) pg/ml] and ANG2 [2062 (1296–3328) pg/ml] expression. Cluster B included 23 patients with poorly vascularised tumours showing lower VEGFR2 [2289 (1577–9776) pg/ml] and ANG2 level expression [709 (4079–1204) pg/ml]. Significant associations were found between the radiomics score and vascularisation clusters showing higher radiomics score in the vascularised cluster A [0.51 (0.37–0.66)] and lower radiomics score in the poorly vascularised cluster B [0.29 (0.21–0.445)] (*p* = 0.01) (Fig. 2c).

## Discussion

The increasing development of targeted therapies for NETs has pointed out the unmet need to define predictive biomarkers of response for improving patient selection. The neoangiogenesis process is a key feature in NET carcinogenesis and the field of new antiangiogenic compound development is still active after sunitinib, the only tyrosine kinase inhibitor utilisable in clinical practice. In this study we explored an imaging phenotype based on radiomics features from baseline CT scans that allows the identification of patients most likely to benefit from antiangiogenic treatment.

The developed radiomics signature outperforms clinical features (including Ki-67 and primary tumour type) for predicting response to treatment (AUC 0.75 vs. 0.57). The radiomics signature consisted of five radiomics features, indicating that patients with more spherical and heterogeneous tumours are more likely to respond to antiangiogenic treatment. Also, more intravenous contrast-enhancing tumours are associated with better response. Therefore, we investigated the association of tumour vascularisation (by means of plasma VEGFR2 and ANG2) and the predictive radiomics score, showing that in the TALENT cohort, the CT-radiomics score was associated with VEGFR2 and ANG2 expression. This suggests that radiomics quantification from baseline CT scans can non-invasively provide valuable information about the tumour vascularisation. The TALENT research group has previously shown that in antiangiogenic pre-treated patients, the plasma proangiogenic profiling (VEGFR2 and ANG2 levels) significantly predicts response in NETs [25]. In the subset of non-pre-treated patients, different mechanisms of vascularisation and activation of alternative signalling pathways could sustain the lack of association between proangiogenic profiling and tumour response [26]. Interestingly, our signature did not show significant accuracy differences between patients who had received previously antiangiogenics or not, which may indicate that the radiomics phenotype captures the tumour characteristics that make it susceptible to antiangiogenic response that includes, but is not limited to, tumour vascularisation.

The capacity of the radiomics signature to predict response was tested in an independent population of patients with pancreatic NETs treated with sunitinib, another multikinase inhibitor with antiangiogenic effects but with different targets and affinity than lenvatinib, with a stable performance of the radiomics signature. The integration of prognostic factors such as Ki-67 index and primary tumour site improved the performance of the radiomics-only signature. However, the combined model (clinical-radiomics) presented a modest performance in the sunitinib population that can be related to the clinical differences between patients receiving standard of care treatment and those within clinical trials.

In previous research, a radiomics model based on the analysis of the entire liver was reported, demonstrating predictive value for response to everolimus in advanced NETs [19]. However, to our knowledge, no previous studies have been reported exploring the role of CT-radiomics in predicting response to antiangiogenic multikinase inhibitors in NETs. Several studies have shown that high-grade NETs present lower density (likely related to a lower intratumoral microvascular density) than low grade NETs [18, 27]. Our study population included mostly grade 1 and 2 NETs, and no significant differences in the tumour enhancement was identified between them (Supplementary Fig. 7). Therefore, the relevance of tumour intensity in the CT-radiomics signature represents most likely a true specific predictive biomarker rather than an indicator of aggressiveness or tumour grade.

Although NETs are often highly-dense tumours in contrast-enhanced CT, usually more conspicuous in the arterial phase of the CT scan, some tumours present different patterns and, as shown in the study population, some tumours are better depicted in the venous phase [28]. In this study, an expert radiologist selected the phase (arterial or venous) of the CT scan where the tumour was best depicted. We also investigated the integration of a multiphase model (combining radiomics data from arterial and venous phases) to address this concern. We demonstrated that including radiomics features from multiple phases did not improve the prediction in our population. Furthermore, we correlated the radiomics score with vascularisation factors (VEGFR2 and ANG2) in the population with concomitant imaging and plasma samples and showed significant associations between the radiomics-score and vascularisation-factor expression.

There are some limitations encountered in this study. First, the population used for the training dataset was tied to the clinical trial population. The test cohort was treated with a different agent, although both are multikinase inhibitors with an antiangiogenic effect, and the population slightly differs clinically from the development cohort (i.e., primary site and grade). This limitation is mainly affecting the performance of the combined radiomics-clinical model in the test dataset. Second, we developed a model using only one acquisition phase selected by an expert radiologist, which can influence the radiomics features. However, we explored the performance of including both phases and it did not improve the prediction capacity and showed higher model overfitting. Third, the images in this study underwent evaluation by a consistent expert radiologist. Nevertheless, it is important to acknowledge that variations in segmentation techniques and observers may introduce biases that could impact the reliability of first-order and textural radiomics features, as well as shape and size metrics [29]. Finally, while we recognise the need for homogeneity in radiomics modelling, achieved by focusing solely on liver metastases, we acknowledge that further exploration and potential validation in larger cohorts encompassing primary tumours and other metastatic sites should be pursued. This will contribute to a more comprehensive understanding of the radiomics-based phenotypes in GEP-NETs.

In conclusion, the GEP-NET phenotype evaluated by means of CT-based radiomics can be a useful non-invasive surrogate of the tumour vasculature and has predictive value of tumour response to antiangiogenic targeted agents. The improved prediction of response to antiangiogenic therapy achieved by combining radiomics with clinical prognostic factors can facilitate medical decision making and optimise treatment outcomes for patients with GEP-NETs. Nevertheless, despite the promising results of this study, further research is necessary in larger, prospective trials for implementation of this tool in clinical practice.

## Supplementary Information

SUPPLEMENTARY MATERIAL

## Data Availability

The raw data of imaging scans analysed in this study are not publicly available due to their containing information, as this would compromise the privacy of research participants. Any queries for data access used in this study should be directed to the corresponding author. The codes can be publicly accessed at https://github.com/radiomicsgroup/TALENT_project. We relied on the open-source software dcm2niix (https://github.com/rordenlab/dcm2niix/) for DICOM conversion and 3D Slicer (www.slicer.org; RRID:SCR_005619) [20] for image annotations and computing.
