# Supplementary material for: Radiomics and outcome prediction to antiangiogenic treatment in advanced gastroenteropancreatic neuroendocrine tumours: findings from the phase II TALENT trial
Source: BJC Rep. 2023 Aug 2;1:9. doi: 10.1038/s44276-023-00010-0 (PMC11523983; doi:10.1038/s44276-023-00010-0)

SUPPLEMENTARY MATERIAL

Supplementary Table 1. Participant centres in the TALENT clinical trial (NCT02678780)

Name of the center, city, country
**1.**Vall Hebron University Hospital and Vall Hebron Institute of Oncology, Barcelona, Spain
**2.**Catalan Institute of Oncology (ICO), L'Hospitalet (Barcelona), Spain
**3.**La Paz University Hospital, Madrid, Spain
**4.**12 de Octubre University Hospital, Madrid, Spain
**5.**MD Anderson Cancer Center, Madrid, Spain
**6.**Ramon y Cajal University Hospital, Madrid, Spain
**7.**Miguel Servet University Hospital, Zaragoza, Spain
**8.**Marques de Valdecilla University Hospital, IDIVAL, Santander, Spain
**9.**Donosti University Hospital, Donosti, Spain
**10.** Central de Asturias University Hospital, Oviedo, Spain
**11.** Hospitales Universitarios Regional y Virgen de la Victoria de Málaga, Málaga, Spain
**12.** European Institute of Oncology, Milan, IEO, IRCCS, Italy
**13.** University Hospital of Modena, Modena, Italy
**14.** AOU Careggi and University of Firenze, Firenze, Italy
**15.** Istituto Oncologico del Mediterraneo, Catania, Italy
**16.** S.C. Sarcomi e Tumori Rari, Istituto Nazionale Tumori, IRCCS, Fondazione “G. Pascale,” Naples, Italy
**17.** Osteoncology and Rare Tumours Center, Istituto Scientifico Romagnolo per lo Studio e la Cura dei Tumori (IRST) IRCCS, Meldola, Italy
**18.** University of Manchester and The Christie NHS Foundation Trust, Manchester, United Kingdom
**19.** Gartnavel Hospital, Beatson Oncology Centre, Glasgow, Scotland
**20.** Medical University of Vienna, Vienna, Austria
**21.** Medical University of Graz, Graz, Austria

Supplementary Table 2. CT acquisition parameters of the development and test cohorts

	Development cohort (N=65)*	Test cohort (N=24)
**Manufacturer - Convolution Kernel**		
**Siemens**	**16 (25)**	**6 (25)**
B20f	1 (6)	1 (17)
B20s	1 (6)	-
B30f	1 (6)	1 (17)
B31f	2 (13)	2 (33)
B31s	-	1 (17)
B41s	2 (13)	-
I30f	8 (50)	1 (17)
I41s	1 (6)	-
**Philips**	**24 (37)**	**14 (58)**
B	22 (92)	14 (100)
A	2 (8)	-
**GE Systems**	**21 (32)**	**2(8)**
STANDARD	17 (81)	2 (100)
SOFT	1 (5)	-
CHEST	3 (14)	-
**TOSHIBA**	**4 (6)**	**2 (8)**
FC02	1 (25)	-
FC03	1 (25)	-
FC08	-	2 (100)
FC13	2 (50)	-
**Kilovoltage (kVp)**		
80	1 (2)	-
100	6 (9)	4 (16)
110	2 (3)	1 (4)
120	51 (78)	19 (80)
130	2 (3)	-
140	3 (5)	-
**Slice Thickness (mm)†**	2[2-3]	2 [2-2.125]
**Pixel Spacing (mm)†**	0.744 [0.702-0.817]	0.782 [0.725-0.816]

Note – Data in parentheses are percentages

† Data are median [interquartile range]

Supplementary Methods. Multiphase model

Liver metastases were segmented in the CT-acquisition phase where the lesions were better depicted with the semi-automatic segmentation tool of Slicer 4.11.0. Phase selection and segmentations were performed by an expert radiologist. For the multi-phase study, images from both phases were registered to correct liver displacements from breathing movement or changes in patients' position. Registration was performed using a four-step registration with plastimatch version 1.8.0. The registration consisted of two rigid translations, one affine registration and a deformable registration (DEMONS). To improve the registration, images were first cropped to the liver area and resampled to a voxel size of 1x1x1 mm^3 using B-spline interpolation. Cropping was performed with a liver-ROI obtained with the semi-automatic liver segmentation tool from NVIDIA Clara AI-AA (AI-Assisted Annotation) extension for 3D Slicer. To avoid changes in voxel data from the deformable registration, the inverted transform was applied to the segmentation for the corresponding phase. Closing and median filters were applied to the segmentations to remove holes. Radiomics features were extracted from both phases using Pyradiomics v3.0.1.

Supplementary Figure 1. Examples of axial contrast-enhanced CT images where the tumours are better depicted in the arterial phase (a and c) and in the venous phase (f and h) of patients treated with lenvatinib and sunitinib. (a-b) A 40-year-old female with an advanced pancreatic NET treated with lenvatinib. An heterogenous liver metastases is better depicted in the arterial phase (a) compared with the venous phase (b). (c-d) A 53-year-old female with an advanced pancreatic NET treated with sunitinib. In this case, the liver metastases can be clearly depicted as an enhancing tumour in the arterial phase (c) but it is hardly appreciable in the venous phase (d). (e-f) A 71-year-old female with an advanced gastrointestinal NET treated with lenvatinib with a liver metastasis better depicted in venous phase (f) than in arterial phase (e) compared with the venous phase (f). (g-h) A 79-year-old female with an advanced pancreatic NET treated with sunitinib. In this case, the liver metastases can be seen in both phases but are much more ill-defined in the arterial phase (g) in comparison with the venous phase (h).

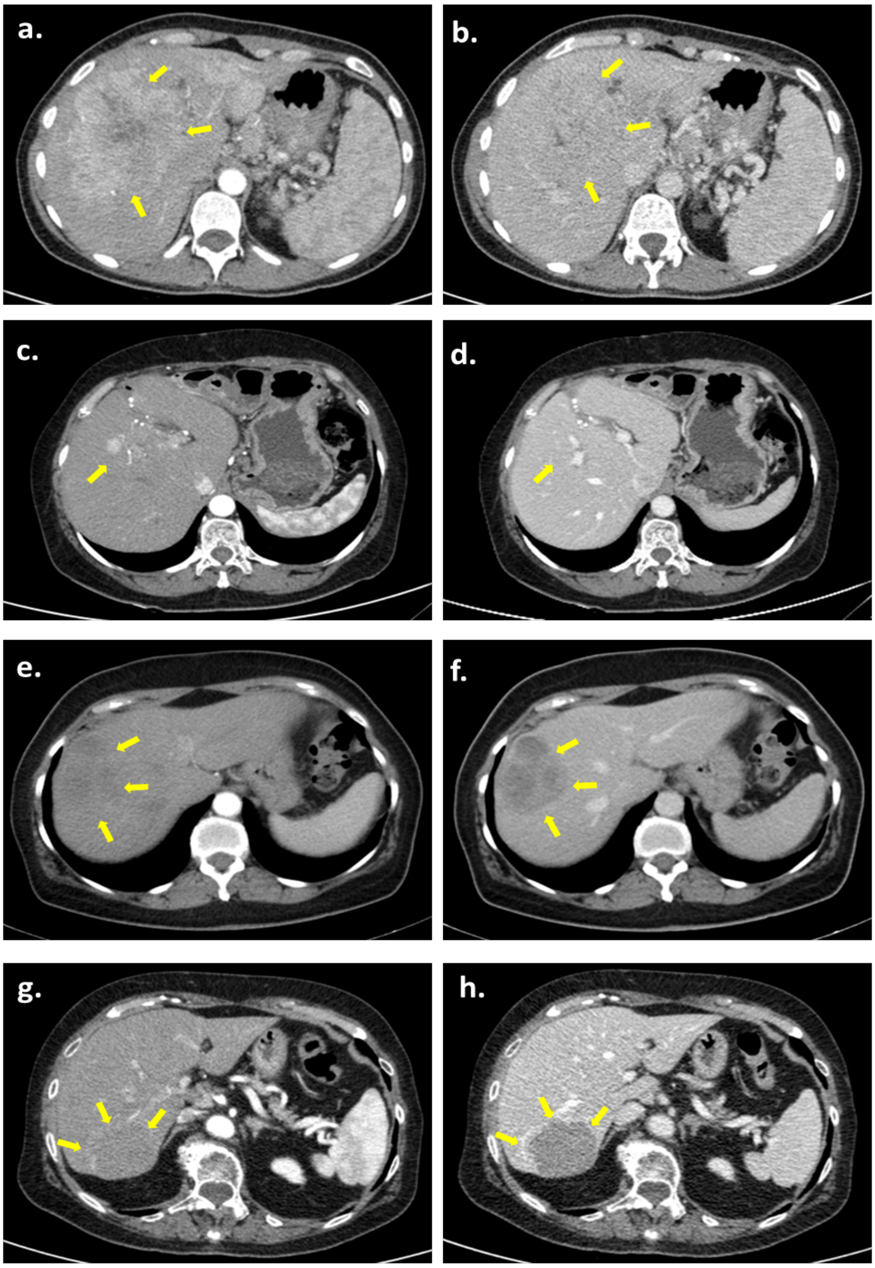

Supplementary Figure 2. Flowchart of the study population. Inclusion and exclusion criteria in the development and test cohorts.

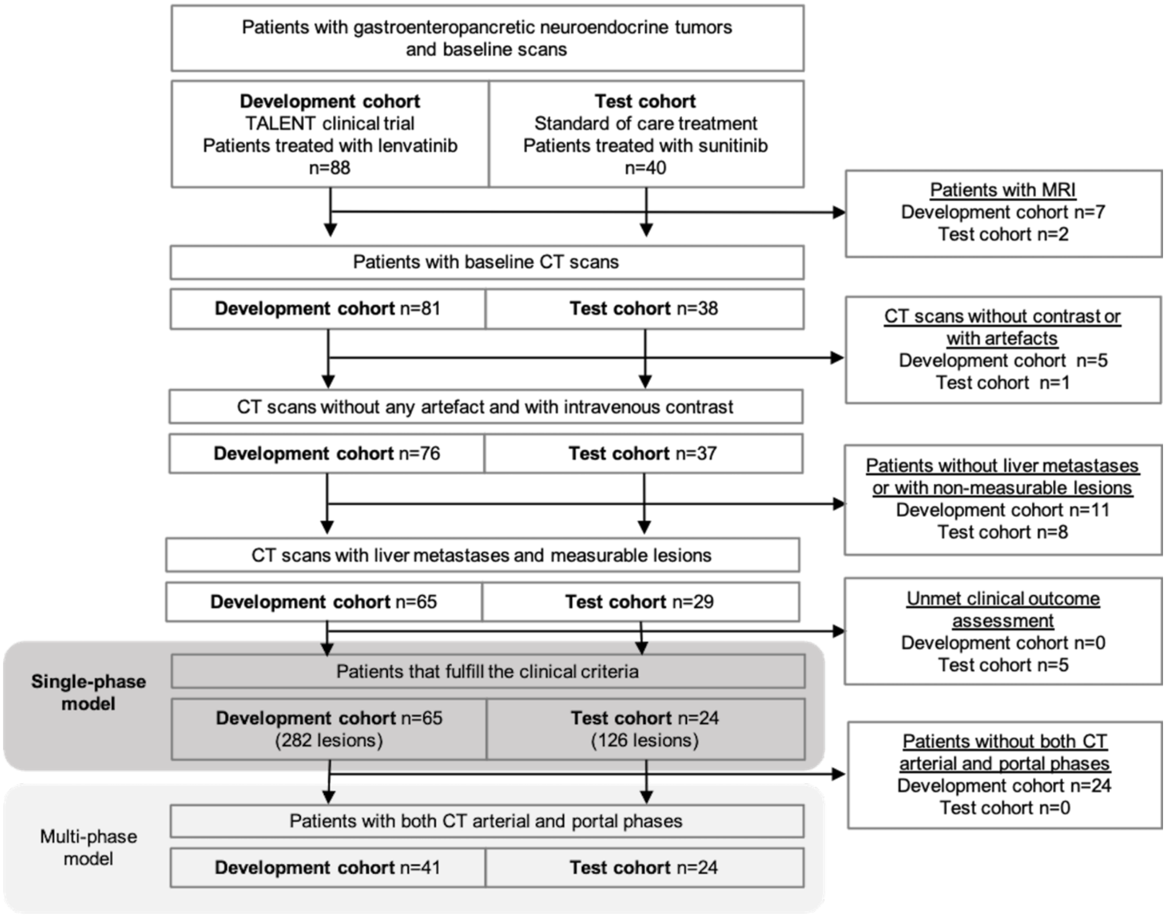

Supplementary Figure 3. K-fold cross-validation repeated 10 times for k=5.

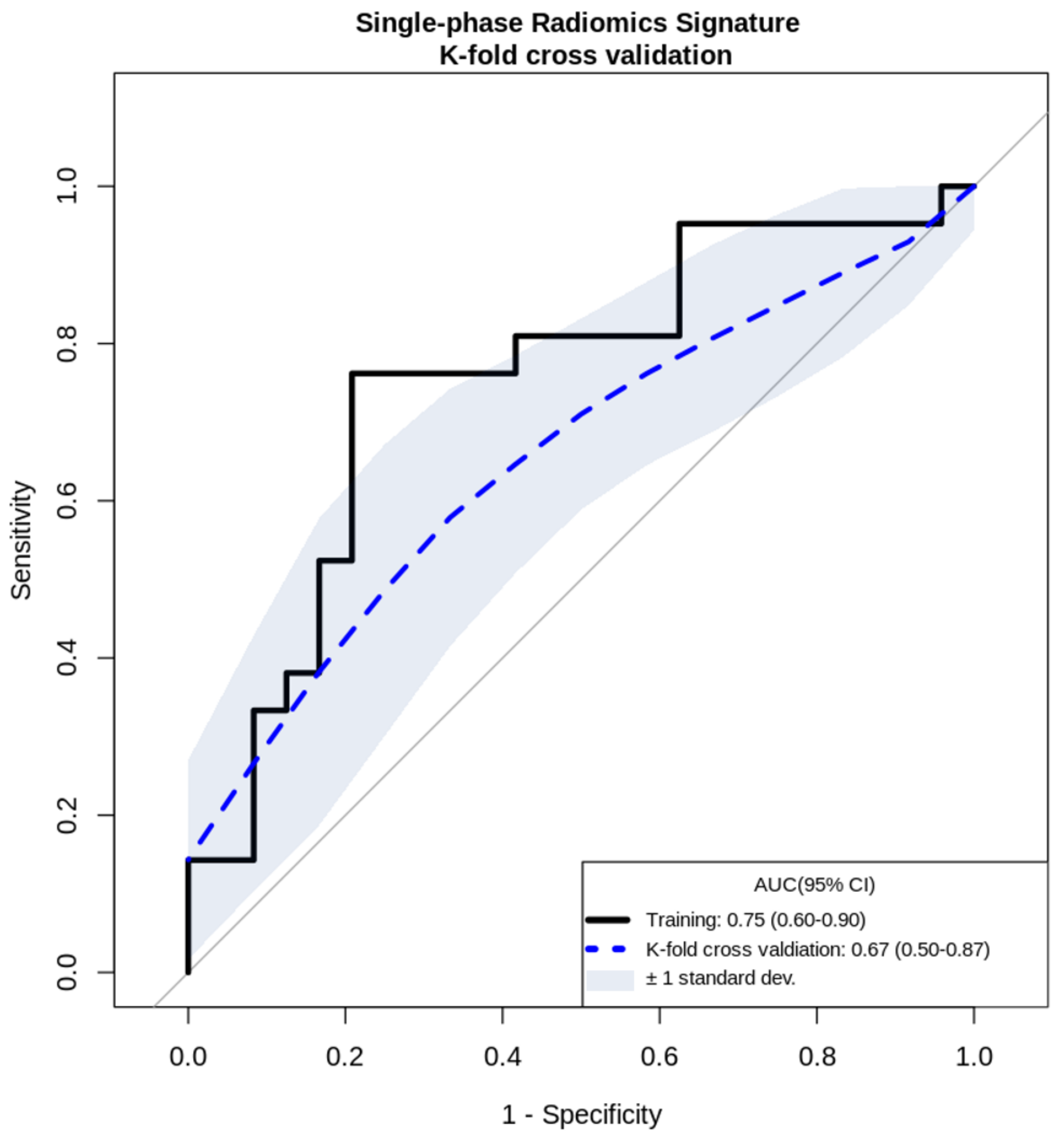

Supplementary Figure 4. Boxplot comparing Radiomics score from pre-treated and non-pretreated patients.

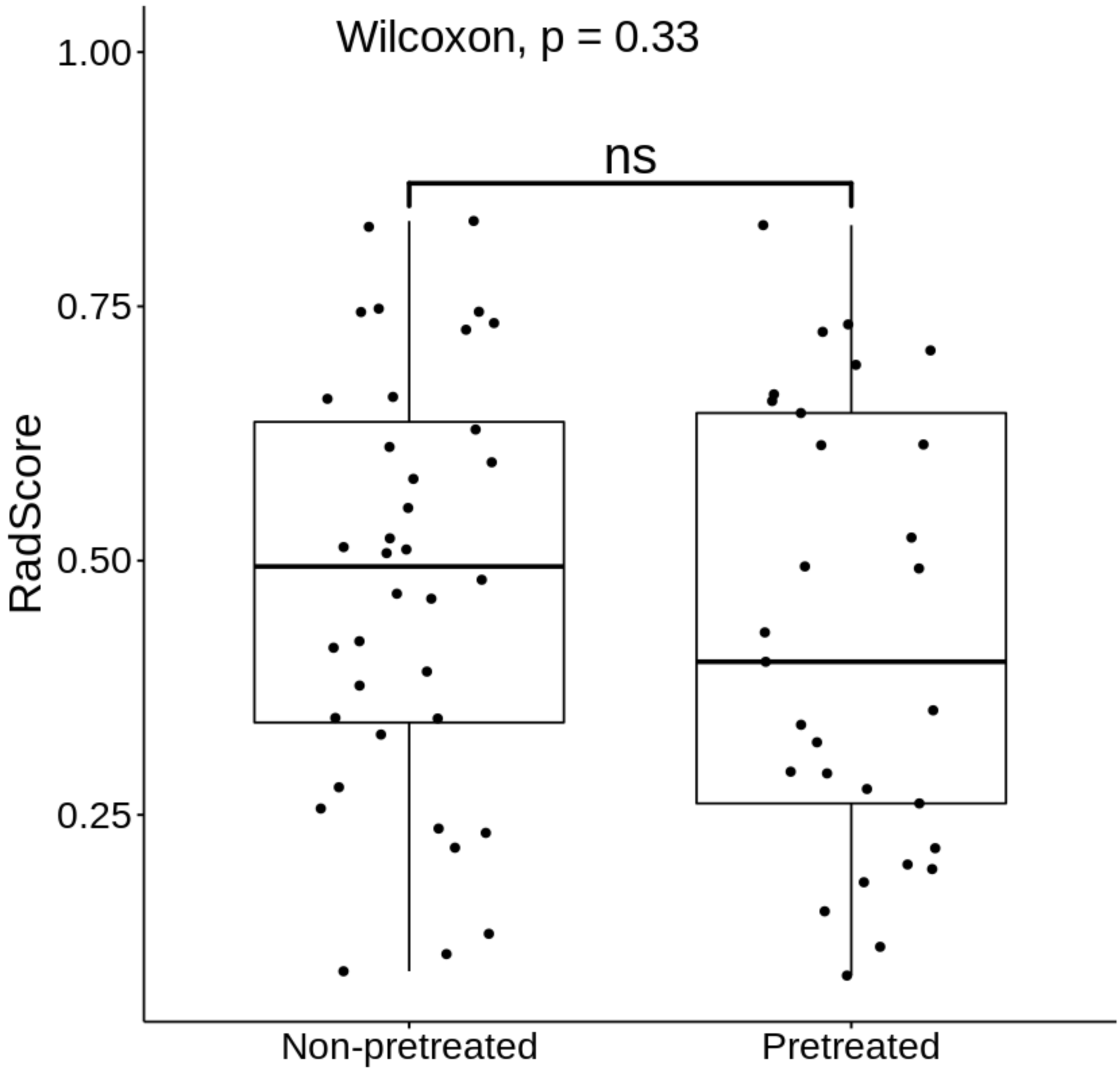

Supplementary Figure 5. ROC curves for the multiphase predictive model combining radiomics features from the CT-scan arterial and venous phases.

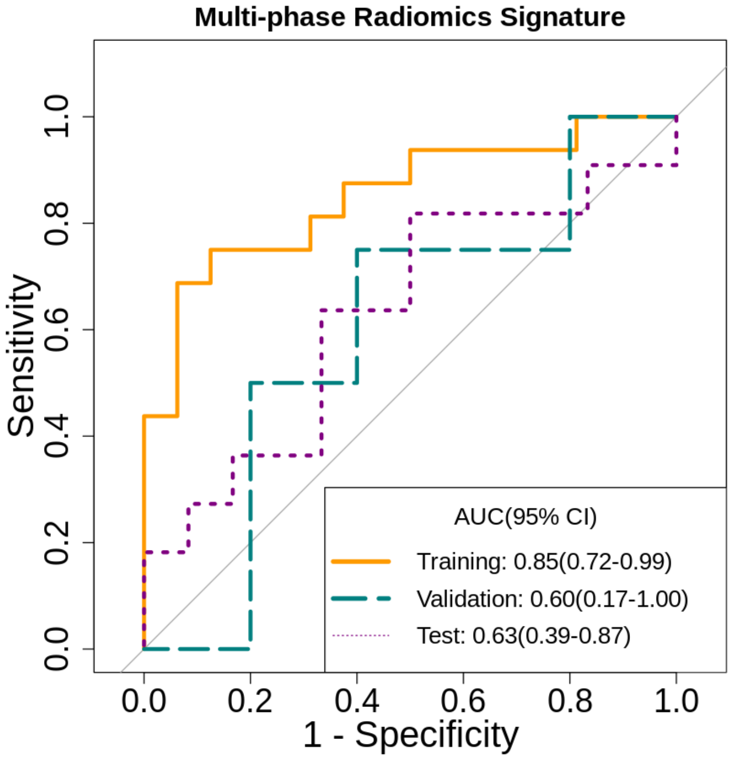

Supplementary Figure 6. Kaplan-Meier curves for progression free survival (PFS) associations with clinical variables (Ki67 range (A) and primary tumour type (B))

A)

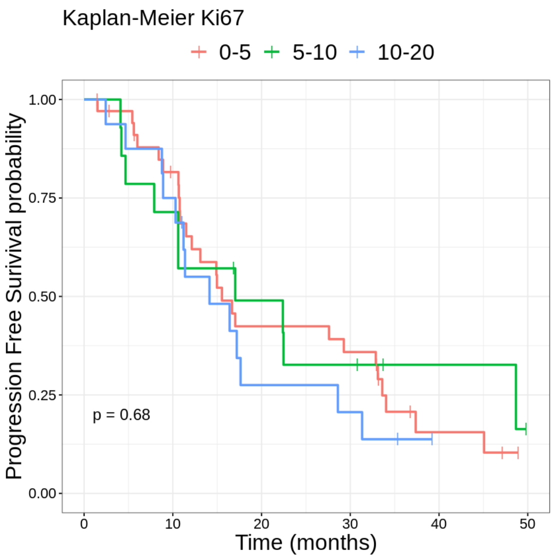

B)

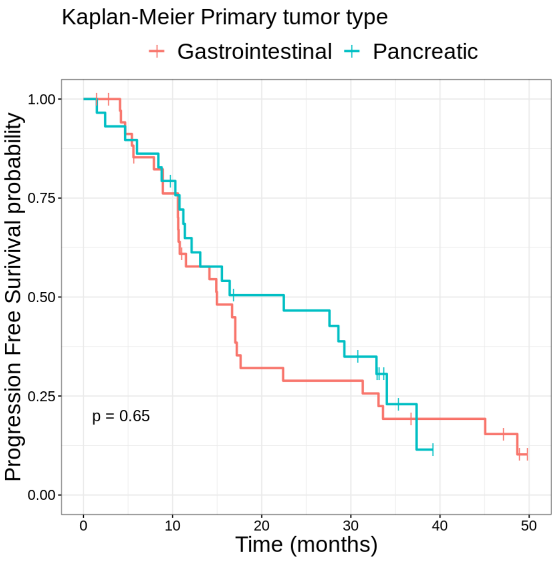

Supplementary Figure 7. Associations between tumour enhancement and neuroendocrine tumour grade for high and low radiomics score. The development and test cohorts are analysed together.

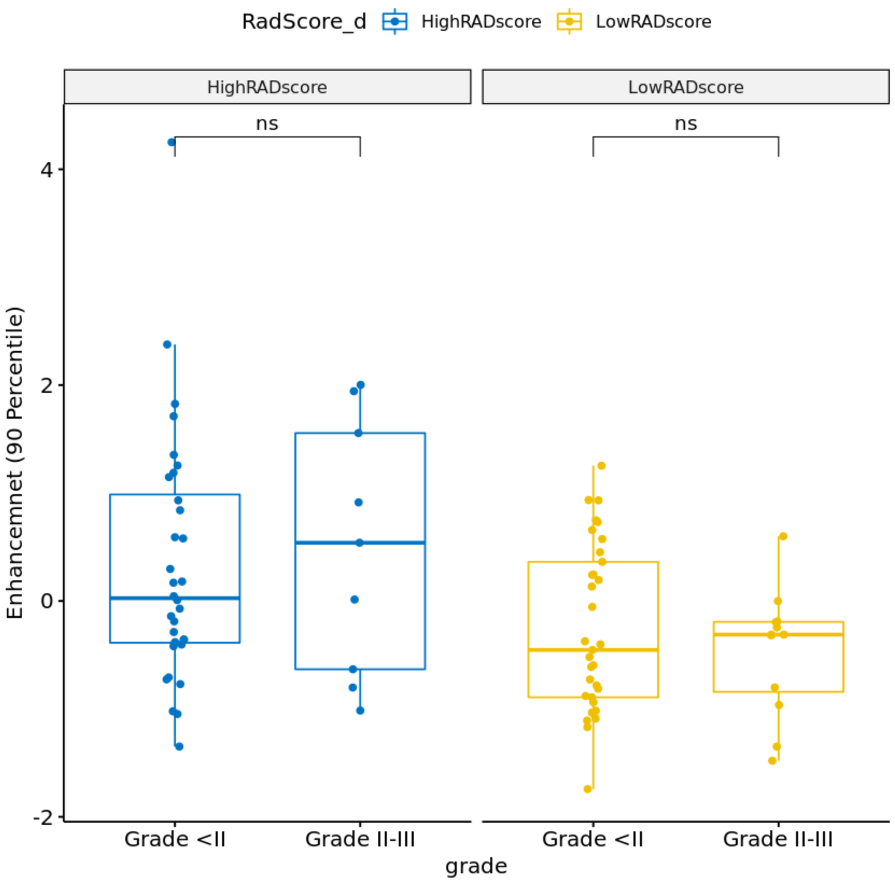

Supplement: Supplementary file 1 — SUPPLEMENTARY MATERIAL [file 44276_2023_10_MOESM1_ESM.pdf]
